# Tracing the early bacterial settlers in preterm and very-low birth-weight infants: first report of microbiota dynamics in South American neonates

**DOI:** 10.1128/iai.00570-25

**Published:** 2026-01-21

**Authors:** Josefina Vera, Catalina Vaz Ferreira, Mario Moraes, Nadia Riera

**Affiliations:** 1 Microbial Genomics Laboratory, Institut Pasteur de Montevideo 123939, Montevideo, Uruguay; 2 Department of Neonatology, Centro Hospitalario Pereira Rossell 601266, Montevideo, Uruguay; 3 Neonatology Academic Unit, Universidad de la República, Centro Hospitalario Pereira Rossell 56724, Montevideo, Uruguay; 4 Center for Innovation in Epidemiological Surveillance, CiVE, Institut Pasteur de Montevideo 123939, Montevideo, Uruguay; University of California San Diego School of Medicine, La Jolla, California, USA

**Keywords:** *Klebsiella pneumoniae*, preterm neonates, microbiome

## Abstract

Mortality in very-low birth-weight (VLBW) infants accounts for up to 50%–70% of the neonatal mortality and up to 25%–30% of infant mortality. Despite the global increase in survival rates, this population remains at heightened risk for developing long-term neurodevelopmental delays, chronic lung disease, malnutrition, and visual and hearing disabilities. The gut microbial composition of VLBW differs from full-term infants and is typically dominated by pathobionts. In this study, we characterized the bacterial composition of the VLBW infant microbiota born at Pereira Rossell Children’s Hospital (academic, tertiary referral center) in Montevideo, Uruguay by sequencing the full-length 16S rRNA gene using Oxford Nanopore Technologies. We describe a high predominance of *Klebsiella pneumoniae* and *Escherichia coli* in these infants. By sequencing stool samples from two time points, we show that the microbial community diversity increases over time with a higher relative abundance of *Bacteroides* and *Veillonella*. Moreover, we describe the effect on the microbial composition of long antibiotic exposure. Different species of the *Klebsiella* genus, along with *Escherichia coli, Enterobacter cloacae*, *Citrobacter freundii,* and *Veillonella parvula* were observed at a higher relative abundance in patients with more than 5 days of antibiotic treatment. Taken together, our findings shed light on the development and establishment of microbial communities in early-life microbial communities in South America. Our results point to postnatal antibiotics as a major factor orchestrating this process. The integration of microbial community health considerations into preterm clinical care is crucial for improving long-term infant development.

## INTRODUCTION

The establishment and maturation of infants' gut microbiota are critical for growth, development, nutrient absorption, metabolism, and disease prevention (1). In the past years, the study of microbiota composition and development in early life has called for attention in the scientific communities, and many studies have emerged aiming to shed light on microbiome composition in large cohorts of term neonates (2–4). Perinatal and early postnatal time points represent the most critical periods for the establishment of the microbiota, being maternal microbiota the main source for infant colonization (5). Factors such as gestational age, mode of delivery (vaginal vs cesarean section), environment, hygienic measures, feeding practices, and antibiotic exposure influence the establishment of microbiota in the perinatal period and are associated with disruption of early colonization patterns leading to dysbiosis in early life (1, 3, 4, 6–8).

Preterm birth (under 37 weeks of gestation) is rising globally, as a consequence of higher maternal age at childbearing and the increase in highly complex assisted fertility treatments. The relevance of very-low birth-weight (VLBW) infant outcomes is that although they represent 1.5% of total births, their outcomes contribute significantly to the neonatal and infant mortality rates. Neonatal mortality rates vary from 4.2 to 18.6 per thousand in South America. Mortality in VLBW infants accounts for up to 50%–70% of the neonatal mortality and up to 25%–30% of infant mortality (9). The survival rate of VLBW infants has increased worldwide as a result of improvement in the quality of prenatal and postnatal care. Despite improved survival rates, complications associated with preterm birth are the leading cause of death among children under 5 years of age throughout the world (6, 9–11). In addition, surviving infants are at significant risk of long-term sequelae, including neurocognitive delays, neurodevelopmental impairment, chronic lung disease (CLD), malnutrition, and visual and hearing disabilities (6, 9, 10).

Neonatal intensive care unit (NICU) hospitalization has shown to severely alter microbial colonization in preterm newborns' microbiota. This altered gut microbiota interaction with an immature immunologic intestinal response triggers proinflammatory and counter-inflammatory cytokine response (6). NICU hospitalization and necessary interventions, including broad-spectrum antibiotics and altered feeding practices that differ from the home environment, can shift the developing gut microbiome dramatically (9). Infants born prematurely typically develop a gut microbial community with low species diversity and high interindividual variation (12). Preterm microbiota patterns differ from their term counterparts who rapidly acquire commensal anaerobes. Instead, preterm infants are initially seeded by nosocomial pathobionts such us staphylococci, enterococci, and *Enterobacteriaceae,* tilting the scale toward a higher risk of bloodstream infections, necrotizing enterocolitis (NEC), among other morbidities and long-term sequelae (5, 7, 13). Antibiotic treatment has a profound impact on the gut microbial community composition of preterm infants (7, 14–16). Most preterm infants receive early and extended periods of antibiotic therapy, with combined therapy of vancomycin and gentamicin being the most common regimen (17). Antibiotic exposure usually results in a marked decrease in the Bifidobacterial population and a community composition governed by Proteobacteria (18). Moreover, antibiotic administration has been linked to an increased abundance of antimicrobial resistance genes (7, 14, 15, 19) and a 10-fold decreased overall bacterial species load (20).

Mother’s own milk (MOM), defined as milk from an infant’s own mother, is a complex combination of nutrients, prebiotics, live microorganisms, and components with antimicrobial and immunomodulatory properties, which plays a crucial role as a supportive and influential factor in the early colonization of the neonatal gut microbiota and in maturation of the immune system (5, 6, 13, 21). The MOM diet has been found to be a major contributor linked to increased species richness over time in preterm infants (20). Human milk oligosaccharides (HMOs) are unique functional components in breast milk and are an important factor in colonization and development of the gut microbiota in preterm infants, acting as prebiotics (13, 21). Recent studies show differences in intestinal microbiota between breastfed and formula-fed infants, with a higher relative abundance in *Bifidobacterium, Staphylococcus, Streptococcus,* and *Lactobacillus* in breastfed infants, whereas formula-fed infants showed a higher relative abundance of *Bacteroides, Clostridium, Enterobacteriaceae, Enterococcus,* and *Lachnospiraceae* (5, 13). For the purpose of this study, we will refer to the term MOM in order to shed light on its specific contribution to shaping infants' gut microbiome. Breast milk, on the other hand, refers to human milk, either pasteurized/donor milk or MOM. Evidence suggests that MOM and donor milk fed infants' gut microbial profiles are different, showing the former a greater presence of *Bifidobacteriaceae* and lower *Staphylococcaceae* (22).

Enhancements in perinatal care should aim to foster targeted strategies to not only decrease preterm birth but also increase survival free of major morbidities. Currently, the development of new therapeutic interventions aimed to modifying the gut microbiota of preterm infants is quickly gaining ground in the scientific community (23), although it is generally accepted that more clinical and preclinical trials are needed. Comprehending the mechanisms governing gut microbiota formation and development in preterm infants is crucial for mitigating morbidity and mortality, fostering their healthy growth. To date, there are no studies of gut microbiota in this vulnerable population in the region, and most of the emerging knowledge arises from northern and developed countries. The aim of this study was to characterize preterm gut microbiota and assess the effect of the mother’s own milk as well as antibiotic exposure in the establishment and development of VLBW infant gut microbiota. In order to do this, we explored the gut microbiota assembly in preterm infants born with less than 1,500 g in a reference pediatric, third-level hospital in Montevideo, Uruguay. We used full-length 16S rRNA (V1-V9) long-read sequencing to explore the bacterial composition at the species level.

## MATERIALS AND METHODS

### Study design

This is an observational, prospective, cohort study of VLBW infants between 24 and 35 weeks of gestation and ≤1,500 g, born at Pereira Rossell Children’s Hospital (academic, tertiary referral center) in Montevideo, Uruguay, between April and October 2024. Infants born with any major congenital abnormality were excluded from the study sample.

The cohort was categorized at two time points: first, at achieving 150 mL/kg/day (T1) and a second sample at 36 weeks’ gestation or at discharge (T2). Infants were allocated to two different groups: either receiving >70% of MOM or fed with less than 70% MOM and other feeding practices. This categorization was made in order to elucidate the impact of MOM in shaping the infants' gut microbiome, compared with infants fed predominantly donor milk (T1) or formula (T2).

Demographic and clinical data of mothers and infants were prospectively collected for both groups (MOM-fed or donor milk/formula-fed). The gestational age (GA) in completed weeks was defined as the best estimate of GA based on the last menstrual period and early prenatal ultrasound. Written informed consent was obtained prior to the sample collection, samples were codified, and data confidentiality was ensured. This study was approved by the Ethics Committee and Institutional Review Board of Pereira Rossell Children’s Hospital, protocol number 8283982. The study was performed in accordance with the Declaration of Helsinki.

### In-hospital mother’s own milk intake

Mother’s own milk is considered the gold standard feeding strategy for all preterm and term neonates; however, barriers to the initiation and maintenance of lactation pose significant challenges to the implementation of this standard of care (24). Collaborative quality improvement strategies have been shown to be an effective tool to improve outcomes in neonatology by systematically applying an evidence-based package of interventions (25, 26). A breast milk quality improvement initiative was performed to optimize MOM feeding. Quality improvement initiatives reveal that systematically implementing practices to support MOM consumption over time leads to an improvement in survival free of major morbidities (27). The protocol consisted of encouragement to initiate colostrum expression following delivery (6–12 h) in accordance with their health status. Moreover, skin-to-skin care was fostered soon after birth as a complementary measure to improve breastfeeding. Expanded and early access to lactation consultants, systematic educational programs were developed targeting health care providers and patients’ families. NICU feeding protocols were updated in order to homogenize practices. A breastfeeding checklist was developed in order to document adherence to the protocol. Registered measures were: time to first colostrum extraction, time from colostrum extraction to administration, average number of the mother’s visiting the lactation room during the first 2 weeks, percentage, and volume of MOM consumption at 7, 14, and 28 days of life and at discharge.

When MOM was not available or did not meet the required daily volume, pasteurized donor milk (mature milk from mothers of term newborns) was administered as a second line feeding strategy during the first 30 days of life (T1). Fortification was started at a 100 ml/kg/day volume of enteral feeds. Beyond the first month of life, preterm formula was administered as a second-line feeding option when MOM was not available (T2). Neither of the infants received probiotics. Vitamin D and iron were supplemented from days of life 8 and 14, respectively, when receiving full enteral feeds.

The proportion of MOM daily consumption was defined as the volume of breast milk intake relative to the total enteral nutrition volume throughout the preceding period to sample collection. An accurate registry of MOM consumption in daily records was fostered and achieved as a collaborative work between NICU nurses and the medical team.

### Fecal sample collection

Fecal sample collection was performed at two time points during hospitalization. The first fecal specimen was obtained at the time of achieving 150 mL/kg/day (T1), and the second sample at 36 weeks of gestation or at hospital discharge (whichever occurred first) (T2). The collection technique consisted of researchers thoroughly washing their hands and wearing hygiene gloves. A sterile swab was used to collect approximately 2–5 g of feces from the diaper and transferred to a sampling tube. Subsequently, the sample was stored in a refrigerator at 4°C for a maximum of 24 h prior to transportation to the Institut Pasteur de Montevideo, where it was initially processed and preserved under 80°C.

### Nucleic acid purification and 16S rRNA gene amplification

Bacterial DNA was extracted and purified using the QIAamp Fast DNA Stool Mini Kit (Qiagen), following a modified protocol optimized for these samples. Briefly, approximately 220 mg of each sample were mixed with 1 mL of InhibitEX Buffer and subjected to vigorous vortexing for 30 min, followed by three cycles of freeze-thaw treatment alternating between –20  °C and room temperature. Samples were then incubated at 70 °C for 5 min, vortexed, and centrifuged. A volume of 600 µL of the resulting supernatant was transferred to a new tube containing 25 µL of proteinase K, and 600 µL of Buffer AL was added, after which the manufacturer’s instructions were followed for the remaining steps of the protocol. The same protocol was performed for the negative control (1 mL of InhibitEX Buffer only) and the positive control (75 µL of ZymoBIOMICS Microbial Community Standard).

Amplification of the 16S rRNA gene was performed using Q5 High-Fidelity 2× Master Mix (New England Biolabs), with primers 27F (5′ TTTCTGTTGGTGCTGATATTGCAGAGTTTGATCMTGGCTCAG 3′) and 1492R (5′ ACTTGCCTGTCGCTCTATCTTCGGTTACCTTGTTACGACTT 3′), both containing Oxford Nanopore-compatible adapters. A total of 2–10  ng of DNA template was used per reaction. Gene amplification was performed with the following cycle: an initial denaturation step of 10 min at 95°C, followed by 25 cycles of denaturation at 95°C for 15 s, annealing at 55°C for 15 s, and extension at 72°C for 30 s.

### Library preparation and sequencing

Library preparation was performed with the Ligation sequencing kit SQK-LSK109 (Oxford Nanopore Technologies) and PCR Barcoding Kit (EXP-PBC096) (Oxford Nanopore Technologies) following the manufacturer’s instructions. In each step, DNA was purified using AMPure XP Reagent beads, and quality and concentration were evaluated using the Qubit 2.0 Fluorometer (Thermo Fisher Scientific) and 1% agarose gel electrophoresis. Sequencing was performed on a GridION x5 platform (Oxford Nanopore Technologies) employing R9.4.1 FLO-MIN106D flow cell. Base calling was performed using Super-accurate base calling (SUP) v3.3.

### Data and statistical analysis

Taxonomic assignment was performed using the open-access software Porefile, an open-access Nextflow pipeline for 16S taxonomic profiling with the SILVA reference database (Silva Release 138.2). Porefile performs a read-by-read taxonomic classification of 16S sequencing data using a mapping strategy against the selected database and utilizes the lower common ancestor (LCA) algorithm implemented in MEGAN6 (28) to assign the read taxonomy (full documentation available at https://github.com/microgenlab/porefile). Subsequent data analysis was conducted in RStudio (v4.4.3), employing packages phyloseq, microbiome, microbial, vegan, readxl, and LEfSe(r) for microbial community profiling, relative abundance estimation, and differential abundance testing among groups. Alpha diversity of the microbial communities was calculated based on four different metrics (Shannon index, Chao1, invSimpson, and Observed richness) using the phyloseq R package.

Ordination plots were calculated based on the Bray-Curtis distance matrix using the Nonmetric Multidimensional Scaling method. Statistical differences among groups were calculated with a Permutational Multivariate Analysis of Variance (PERMANOVA) analysis using the vegan package (permutations = 999). Differential expression of species was performed using the microbial R package with an adjusted *P* value of 0.001 and log2 fold change of 5. The biomarker identification was calculated using the microbial R package.

## RESULTS

### Characteristics of the general study population

A total of 20 VLBW infants were included in the study. Regarding prenatal characteristics, the mean maternal age and body mass index were 26.9 ± 1.9 years and 22.3 ± 5.5, respectively. Gestational diabetes and pregnancy hypertensive disorders were present at 25% and 40% of the cases; 75% of the pregnancies received antenatal steroids, 37% intrapartum antibiotics, and 40% of the deliveries were vaginal. Considering the neonatal characteristics of the cohort, the mean birth weight and gestational age were 1,210 ± 238 g and 29.9 ± 1.9 weeks, respectively; 60% of VLBW were female sex. The mean days at T1 were 14 ± 11 days. Regarding feeding practices, 75% of the infants received their mother’s colostrum in the first 72 h of life. At the time of the first sample (T1), 25% of them were feeding more than 70% of MOM, and 75% received predominantly donor milk. At the time of sample 2 (T2), 15% were feeding more than 70% of MOM, and 85% were predominantly formula fed. The cohort received postnatal antibiotics in 85% of the cases with a mean of 7.9 ± 10.2 days of exposure. Regarding neonatal morbidities, 5% presented late-onset sepsis, 0% necrotizing enterocolitis and severe intraventricular hemorrhage (grade III–IV), and 20% bronchopulmonary dysplasia and retinopathy of prematurity. Mean hospital stay was 62.6 ± 28.1 days (Table 1).

**TABLE 1:** General study population (*N* = 20)^*a*^

Prenatal characteristics	
Maternal age (years); mean ± SD	26.9 ± 1.9
Primiparous %	50
Body mass index	22.3 ± 5.5
Gestational diabetes %	25
Chorioamnionitis %	0
Pregnancy hypertensive disorders %	40
SGA %	35
Antenatal steroids %	75
Vaginal delivery %	40
Intrapartum antibiotics %	37
Neonatal Characteristics	
Birth weight (g) (mean ± SD)	1,210 ± 238
GA (weeks) (mean ± SD)	29.9 ± 1.9
Days at T1 (mean ± SD)	14 ± 11
Female sex %	60
Colostrum in first 72 h %	75
> 70% MOM at T1 %	25
> 70% MOM at T2 %	15
Antibiotic exposure (days) (mean ± SD)	7.9 ± 10.2
Postnatal antibiotic exposure %	85
NEC %	0
LOS %	5
BPD %	20
Grade III–IV IVH %	0
ROP %	20
Hospital stay (days) (mean ± SD)	62.6 ± 28.1

^*a*^ GA, gestational age; T1, time 1; SGA, small for gestational age; MOM, mother's own milk; NEC, Necrotizing enterocolitis; LOS, late onset sepsis; BPD, bronchopulmonary dysplasia; IVH, intraventricular hemorrhage; and ROP, retinopathy of prematurity.

### *K. pneumoniae* and *E. coli* dominate the gut microbial composition in VLBW infants

**Fig 1:**
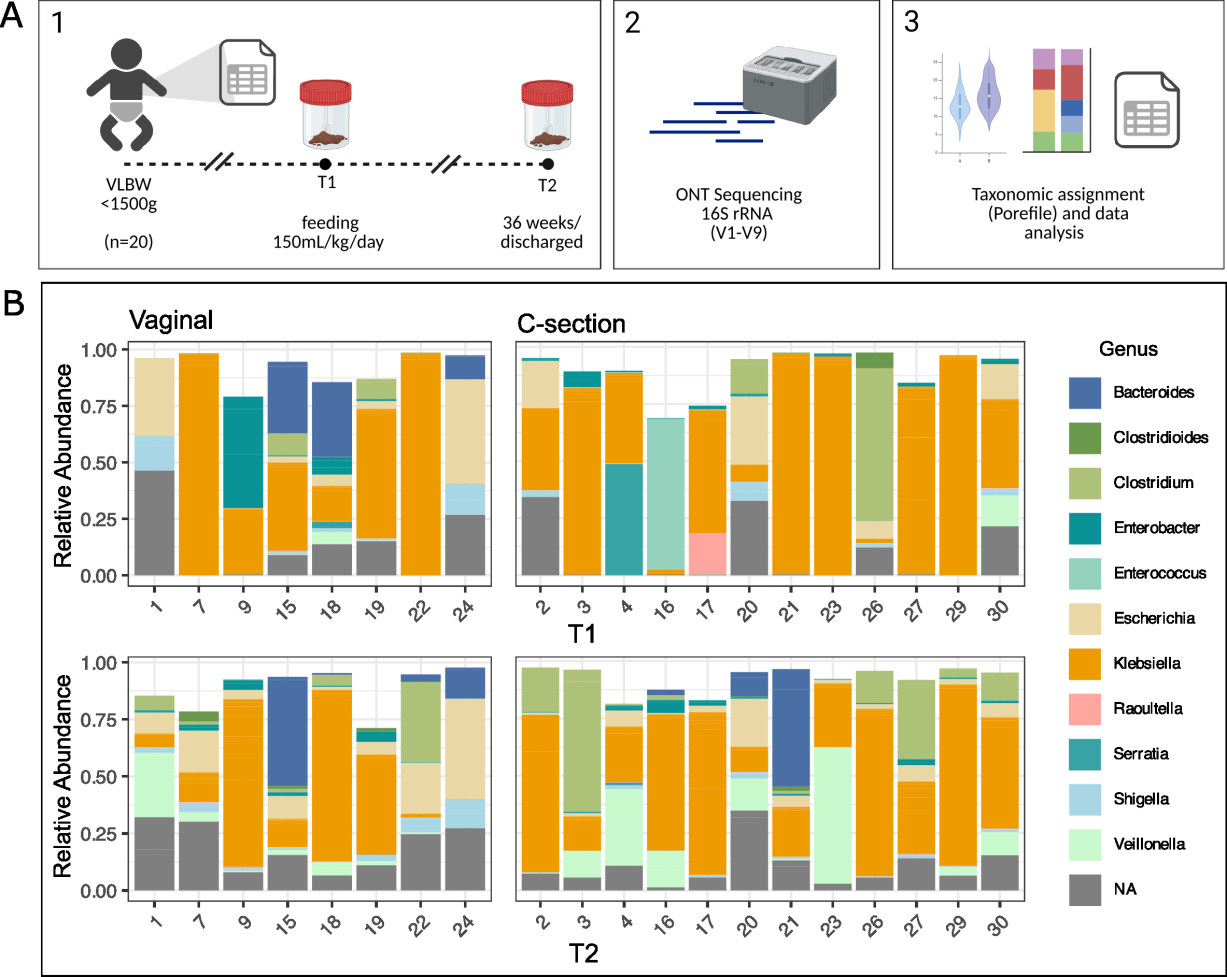
Relative abundance of the most represented bacterial genus in the VLBW preterm infant gut. (**A**) Schema of sample collection, sequencing, and data analysis. (**B**) Taxonomic composition (genus-level) was measured as relative abundance per patient in vaginal and cesarean delivery.

Recent advances in sequencing technologies and the use of complete 16S sequences (V1–V9) from long-read technologies often allow for species-level resolution (29). Using sample preparation and data analysis, the microbial profiling for each VLBW infant was compared at T1 and 36 weeks GA/discharge (T2) (Fig. 1A). We observed that independent of the mode of delivery, a strong dominance of *Klebsiella* genus was present in most of the VLBW infants of the cohort (Fig. 1B). Additionally, in almost half of the study population, a high relative abundance of the genus *Escherichia* was also observed. In almost all samples at birth (18/20 of T1), the relative abundance of *Klebsiella* and *Escherichia* explained more than 50% of the population (Fig. 1B). At the species level, *K. pneumoniae* species complex (*Klebsiella variicola*, *Klebsiella quasivariicola*, and *Klebsiella quasipneumoniae*) and *Klebsiella oxytoca* species complex (*Klebsiella oxytoca* and *Klebsiella michiganensis*) together with *E. coli* explained more than 50% of the total microbiota composition of VLBW infants (see Fig. S1 at https://doi.org/10.6084/m9.figshare.30661181). Only two infants presented a high relative abundance of *Enterococcus* (Patient 16) or *Clostridium* (Patient 26), which explained 70% of the bacterial population.

### Microbial composition changes over time in the VLBW microbiome with an increased species richness

**Fig 2:**
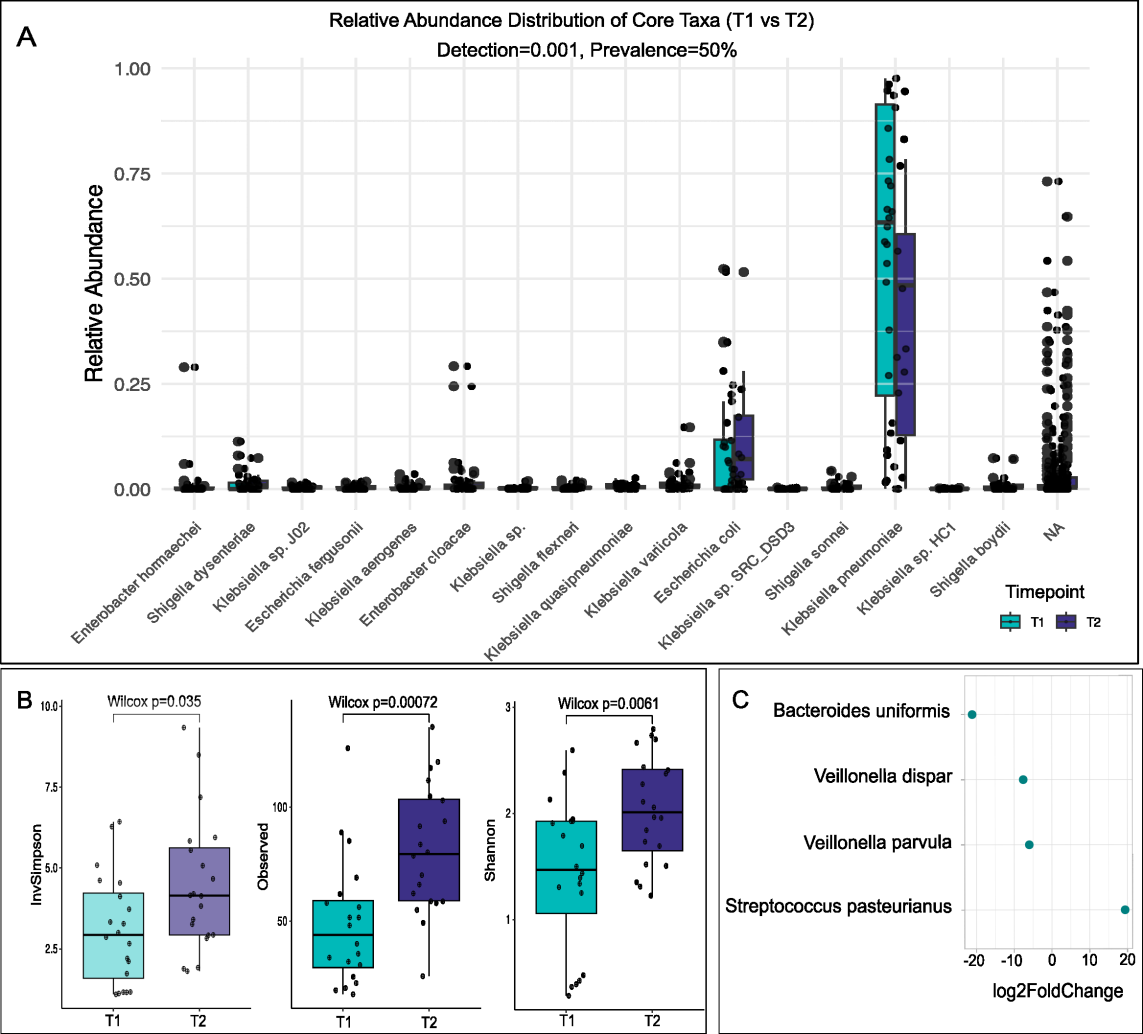
Bacterial community composition changes over time in VLBW infant gut microbiome. (**A**) Relative abundance of core taxa present in VLBW infants in T1 and T2. (**B**) Increase in bacterial diversity as measured by alpha diversity. (**C**) Log2-fold change with significant biomarkers in T1 and T2.

In order to study the dynamics of the microbial composition of VLBW, we characterized the taxonomic profile and the relative abundance of the most prevalent taxa. By comparing the gut microbiome at the initial (T1) time point with the 36-week GA/discharge (T2), we identified bacterial changes in the community structure. Firstly, we calculated the core taxa as those bacterial species present in more than 50% of the samples with a relative abundance of at least 0.01%, and we observed changes in the bacterial composition in T1 and T2 (Fig. 2A). Based on these parameters, we identified *K. pneumoniae* and *E. coli* as the core taxa with higher relative abundance in our cohort. Secondly, we sought to explore the dynamics of the accessory taxa of these preterm babies for the two time points. We defined accessory taxa as those with a prevalence higher than 20% but below 50% and with the relative abundance threshold of 0.01%. We obtained a total of 42 taxa that fulfilled these requirements, of which 10 were classified as different species of *Klebsiella* sp., further supporting the strong dominance of this genus as an early colonizer of preterm VLBW infants (see Fig. S2A at https://doi.org/10.6084/m9.figshare.30661181). Although not widespread, taxa from pathogenic species such as *Citrobacter freundii*, *Citrobacter koseri*, *Enterobacter* sp., *Salmonella enterica*, and *Burkholderia pseudomallei* were frequent early colonizers in this vulnerable population (see Fig. S2A at https://doi.org/10.6084/m9.figshare.30661181). As expected, the bacterial diversity measure as alpha diversity consistently increased in the second time point for the VLBW infants (Fig. 2B). We were not able to see significant changes in the microbial composition based on Bray–Curtis distance matrix (PERMANOVA, *P* = 0.235) for the variable time (T1 vs T2) (see Fig. S2B at https://doi.org/10.6084/m9.figshare.30661181). Similarly, no significant compositional changes were observed across the other clinical variables explored (see Fig. S3 at https://doi.org/10.6084/m9.figshare.30661181). Next, we calculated the differential expression of bacterial taxa in the samples and measured the log2-fold change between the time points. We observed that *Bacteroides uniformis*, *Veillonella dispar,* and the *Veillonella* genus were overrepresented in T2, whereas *Streptococcus pasteurianus* was differentially found in T1 (padj = 0.001 and log2FC = 7). Collectively, after adjusting for delivery mode and feeding practices, our data show an increase in the microbial diversity over time with an increased relative abundance of the genus *Veillonella* and *Bacteroides* (Fig. 2C). We found no significant associations of the documented clinical variables with the microbial relative abundance of bacteria (see Fig. S4 at https://doi.org/10.6084/m9.figshare.30661181). Further studies with more data of the preterm microbiome are needed to evaluate these associations.

### Days of antibiotic exposure significantly affect the microbial diversity in VLBW

**Fig 3:**
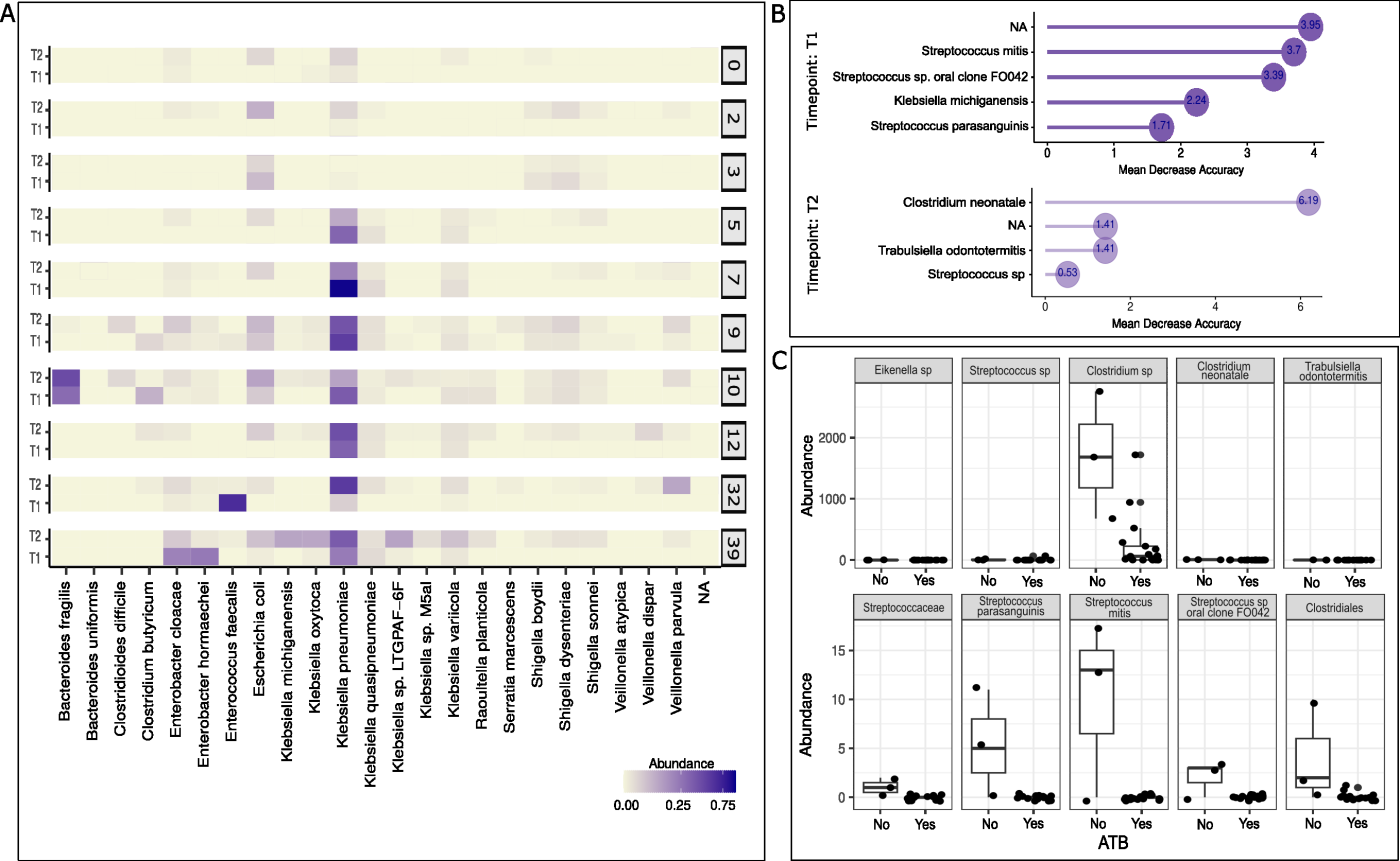
Days of antibiotic exposure impact on microbial abundance of VLBW infants. (**A**) Species abundance faceted by the days of antibiotic exposure in very-low birth-weight infants (top species are represented, scale sqrt). (**B**) Biomarker identification of bacteria in T1 and T2 (biomarker R package), (**C**) Relative abundance of species identified as potential biomarkers for T1 and T2.

Very-low birth-weight infants often spend prolonged periods of hospitalization before discharge and are over-exposed to clinical interventions compared to their full-term counterparts. During this window of extreme vulnerability, antibiotic treatment is frequently administered during several days as a way to mitigate infections and elude further complications. All antibiotics administered in this cohort were broad-spectrum antibiotics (see Table S1 at https://doi.org/10.6084/m9.figshare.30661181). As expected, we identified a strong impact of days of antibiotics treatment on the microbial composition of newborns. A strong signal of *K. pneumoniae* relative abundance in the gut microbiome was found in newborns with more than 5 days of antibiotic treatment (Fig. 3A). In addition to *K. pneumoniae*, the relative abundance of *Klebsiella quiasipneumonieae* and *K. variicola*, members of the *K. pneumoniae* Species Complex, was also increased after 5 days of subsequent antibiotic interventions and not before. Interestingly, *E. coli* relative abundance remained relatively constant even at prolonged periods of antibiotic treatment (12, 32, and 39 days). *Enterobacter cloacae*, *Citrobacter freundii,* and *Veillonella parvula* were also observed at a higher relative abundance in patients with more than 5 days of antibiotic treatment (Fig. 3A). We sought to identify which microbial biomarkers could serve as features to discriminate between antibiotic-treated and non-treated VLBW. We identified *Clostridium*, *Trabulsiella,* and *Streptococcus* as potential biomarkers based on Random Forest estimation in T2 and *Streptoccoccus* and *Klebsiella* in T1 (Fig. 3B and C). The mean decrease accuracy predicts those bacterial markers as the most relevant features discriminating one group or the other for T1 and T2, respectively. Next, we compared the microbial composition between antibiotic-treated and non-treated patients. As expected, the bacterial community was significantly different between groups based on the Bray-Curtis distance matrix (PERMANOVA, R2 = 0.078 and *P* = 0.005) (Fig. 3D). Taken together, these results show that antibiotic exposure causes a strong perturbation of the microbial community in the preterm infant’s gut microbiome.

### Effect of MOM diet in VLBW preterm infants

**Fig 4:**
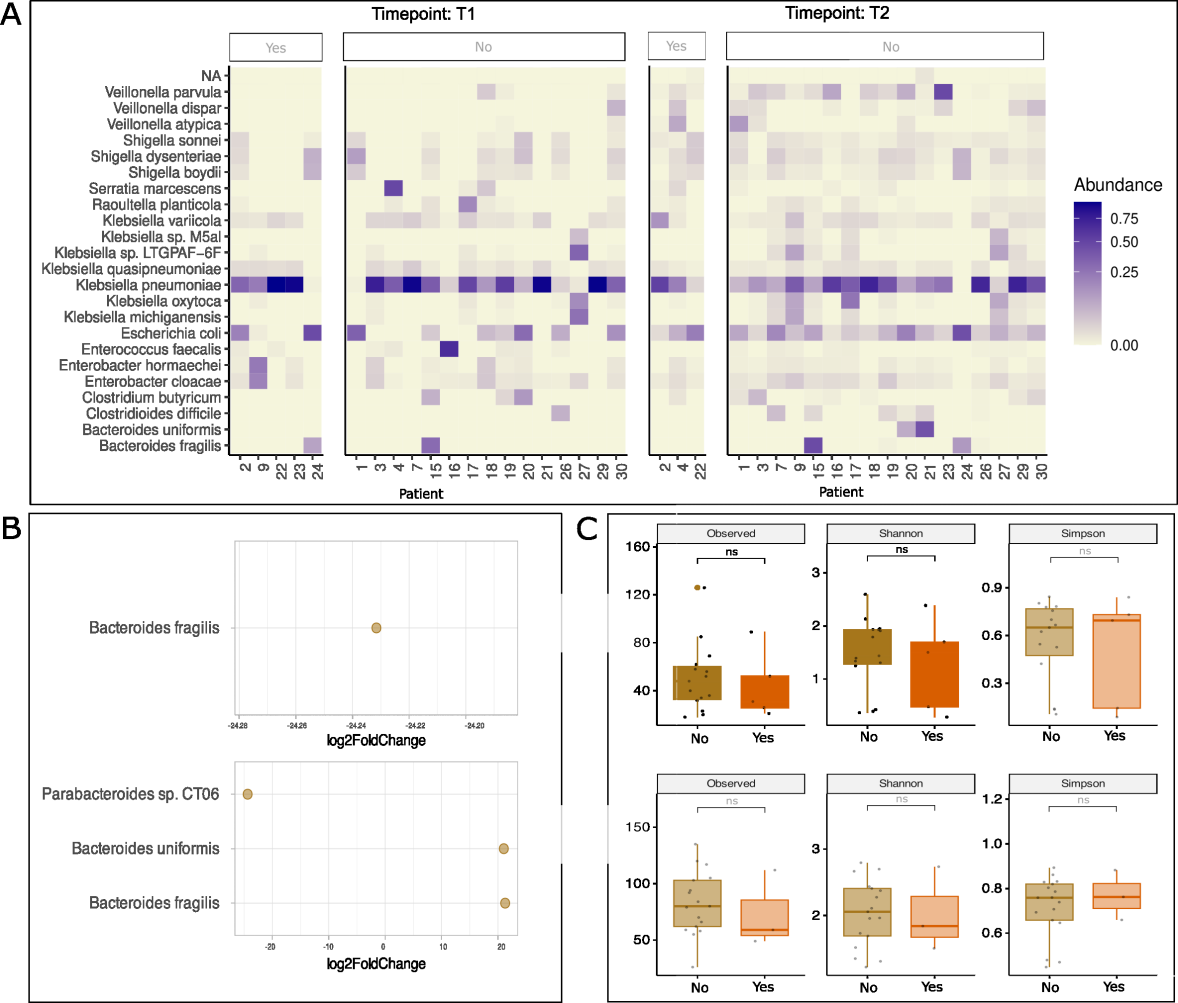
Effect of different dietary interventions on microbial composition (MOM > 70% vs MOM < 70%). (**A**) Top 30 most abundant species in MOM > 70% (yes) vs MOM < 70% (no) VLBW infants in T1 and T2. (**B**) Differential expression of species in MOM > 70% vs MOM < 70% (adjusted *P* value of 0.001 and log2-fold change of 5) in T1 (top) and T2 (bottom). (**C**) Bacterial diversity as measured by alpha diversity (observed richness, Shannon, and Simpson indices) in T1 (top) and T2 (bottom). ns, not significant.

In order to study the impact of the MOM diet on microbiome assembly, the breastfeeding quality improvement program was implemented. Detailed practices of the program including early colostrum administration, mothers’ own milk, fostering breast milk-maintained extraction, and early fortification programs (80–100 mL/kg/day) are all documented in Materials and Methods. Importantly, when MOM was not available or did not meet the required daily volume, pasteurized donor milk was administered as a second-line feeding strategy during the first 30 days of life (T1). In our cohort, five infants were mostly fed with MOM (>70%) at time T1, whereas 15 were fed predominantly with donor milk at time T1. Only three remained with this high proportion of maternal diet at T2; the remaining 17 were predominantly formula-fed. We did not find significant differences in the alpha diversity between predominantly MOM-fed infants as opposed to donor milk-fed in T1 and formula-fed in T2 (Fig. 4).

## DISCUSSION

The data presented provide new insights into the microbial community structure in the preterm gut in a public, third-level reference hospital in South America. We performed a longitudinal study of 20 VLBW infants in order to characterize and evaluate in-hospital mothers’ own milk intake, as well as other variables, on the development of gut microbiota. Earlier studies on the microbial composition of preterm infants often relied on analyzing a small portion of the 16S rRNA gene using second-generation sequencing (30). This approach, based on short reads, is generally used to characterize the bacterial community at the genus level but is typically insufficient to classify the reads at the species level. In this study, using full-length amplicons of 16S (regions V1–V9) sequences in combination with Oxford Nanopore technologies (ONT), we were able to describe the community composition of VLBW infants at high resolution and observed a high predominance of *K. pneumoniae* dominating the gut microbiome independent of the mode of delivery (Fig. 1; see Fig. S1 at https://doi.org/10.6084/m9.figshare.30661181). In contrast with full-term infants who usually present differential patterns in vaginal and cesarean delivery (4, 31), preterm infants’ microbiome has been associated with lower bacterial colonization and an establishment independent of the mode of delivery (20). High prevalence of the genus *Klebsiella* in the preterm gut microbiome has been previously explored (32, 33), and many of these studies have focused on NEC-affected infants (30, 34, 35). In a study of 60 VLBW infants, the authors reported an over-time increase in the relative abundance of *Escherichia-Shigella* for both human milk-fed and formula-fed infants (21).

Regarding the two infants with a high relative abundance of *Enterococcus* (Patient 16) and *Clostridium* (Patient 26) explaining 70% of the bacterial population, we believe that certain clinical characteristics could, partly, explain their being different from the rest of the cohort. Patient 16 was a very sick patient from the first days of life, received multiple and prolonged courses of antibiotic treatment, was kept on minimal enteral feeds during the first month, and presented with necrotizing enterocolitis-like symptoms; however, the patient was not diagnosed with this condition. Patient 26 was found to have an obese mother; nevertheless, no particular known clinical feature that can be explicative of its high relative abundance of *Clostridium*.

An altered microbial colonization can act as a major contributor to the development of impaired intestinal barrier function, metabolic and inflammatory diseases, as well as an increased risk of developing neurological diseases (36–38). Thus, understanding the microbial composition assembly in preterm infants and its clinical drivers is a priority to incorporate microbiome-based treatments in the future.

Previous reports indicate an exclusionary competition where *Klebsiella* outcompetes *E. coli* in the gut microbiome of newborns affected by necrotizing enterocolitis (NEC) (32). The etiology of this pathology, although not fully understood, presumably involves the activation of the Toll-like receptor 4 (TLR4) signaling pathway by bacterial lipopolysaccharides (LPS) (39, 40). Our cohort notably demonstrated that most infants were able to concurrently harbor both *E. coli* and *Klebsiella* in their gut microbiomes (see Fig. S1 at https://doi.org/10.6084/m9.figshare.30661181). This co-occurrence aligns with the low incidence of NEC observed in this cohort, as none of these infants developed the condition. Indeed, the NEC incidence at the Pereira Rossell Hospital was 5% in 2024, lower than average in the region (9%). Further studies are needed to understand the complex interplay between *Klebsiella* and *E. coli* and their role in the establishment of the gut microbiome in the immature gut of preterm infants.

The preterm gut environment, often subject to extensive and prolonged antibiotic exposure, presents a significant challenge to microbiome establishment (17). We sought to understand the impact of prolonged antibiotic treatment in our cohort. As expected, we observed a significant change in the microbial composition in antibiotic-treated and non-treated patients (Fig. 3). Specifically, infants exposed to at least 5 days of antibiotic exposure present a strong shift in the relative abundance of *K. pneumoniae, K. variicola, K. aerogenes, and K. michiganensis*. Our findings are in accordance with recent reports showing minimal immediate antibiotic effects; however, suggesting more pronounced shifts emerging over extended periods of antibiotic exposure (19).

In a typical maturation of the gut microbiome in full-term infants, facultative anaerobes can appear as early colonizers, and progressively strict anaerobes start to proliferate as oxygen concentrations decrease (reviewed in (20). Antibiotics not only impact the microbial composition due to their direct antimicrobial activity but also can increase oxygen levels in the gut lumen by diffusion from the host bloodstream (41). In our cohort, we observed an increase in the relative abundance of *Bacteroides* and *Veillonella* genera at T2, both of which are strictly anaerobic (Fig. 2). This suggests a complex dynamic and assembly in preterm neonates, where, despite the early shifts favoring facultative anaerobes, some anaerobic groups can still proliferate and colonize in later stages of colonization. This complex interplay should be further studied in VLBW infants, who have to undergo the pressing effort of increasing brain size development outside the maternal protective environment. In fact, the third trimester of pregnancy is critical for brain growth and function (42, 43), and the implications of navigating this neurodevelopmental stage with a severely impaired microbiome should be further considered in the context of the gut-brain bidirectional communication.

Recently, low bacterial diversity has been proposed as a predictive biomarker of NEC (7). We hypothesize that MOM and donor milk from the human milk bank available at the Pereira Rossell Children’s Hospital may act as a prebiotic factor that may account, at least in part, for the protective microbiome effect seen in our population. Current evidence shows that HMOs from donor milk remain mostly unchanged after holder pasteurization. Donor’s milk diet in VLBW can act as a prebiotic and exert a range of effects such as antimicrobial activity, modulation of the intestinal epithelial cell immune response, and promotion of growth of commensal bacteria (44, 45). Human milk has been shown to reduce NEC incidence as well as late-onset sepsis, promoting a healthier brain development (46). We believe that not having found statistically significant differences in feeding practices can be explained by the fact that at T1, 25% of the cohort received a predominantly MOM diet compared with 75% fed donor milk predominantly. On the other hand, at T2, only 15% of the infants received a predominantly MOM diet, consequently masking the potential differences.

This study has limitations authors acknowledge. First, the study sample size of VLBW is small, which can lead to potential bias in statistical power. Second, neither did we conduct microbial testing for breast milk or formula nor perform vaginal-swab testing prior to preterm delivery. Finally, in this study, only the bacterial component of the microbial community was studied. Further studies are needed to understand the role of the complex community of yeast, archaea, and viral components in the colonization and establishment of the microbial community. We believe that the main strength of this study lies in being the first VLBW infant gut microbiome characterization study of the region, both exploring the impact of the MOM diet on its development and fostering a quality improvement initiative in order to improve MOM use in this vulnerable population.

### Conclusions

Collectively, these findings support the idea that the development and evolution of early-life microbial communities in the VLBW infant’s gut is a continuous, not random process, and that breast milk, postnatal antibiotics, and the NICU setting bear an influence on this process. Efforts toward incorporating microbial community structure health in preterm clinical care can have a protective long-term impact on infants’ development. We are increasingly acknowledging the role of microbial colonization in long-term health, including gut barrier function, immune modulation, as well as metabolic and neurodevelopmental disorders (including cerebral palsy, autism spectrum disorders, anxiety, and learning disabilities). In this way, efforts should be directed toward understanding which clinical practices and microbiome-based interventions can mitigate the detrimental effect on microbial community structure of prolonged NICU hospitalizations. In this study, we showed how 16S full-length Oxford Nanopore sequencing can be used to describe the bacterial composition at a species level for this vulnerable population in Montevideo, Uruguay.

## Data Availability

All data obtained and reported in this cohort is available at NCBI in BioProject PRJNA1281704. This includes raw 16S rRNA gene sequencing reads with the corresponding clinical metadata, which is available upon request. Raw reads for the positive and negative controls are also available.
